# A cross-sectional study of financial distress in persons with multimorbidity

**DOI:** 10.1016/j.pmedr.2021.101464

**Published:** 2021-06-25

**Authors:** Steven S. Coughlin, Biplab Datta, Adam Berman, Christos Hatzigeorgiou

**Affiliations:** a Department of Population Health Sciences, Augusta University, 1120 15^th^ Street, Augusta, GA 30912, USA; b Institute of Public and Preventive Health, Augusta University, Augusta, GA, USA; c Division of Cardiology, Augusta University, 1120 15th Street, Augusta, GA 30912, USA; d Division of General Internal Medicine, Augusta University, 1120 15^th^ Street, Augusta, GA 30912, USA

**Keywords:** Cancer, Costs, Cardiovascular disease, Financial distress, Multimorbidity, Social determinants of health

## Abstract

**Background:**

Financial distress among persons with multimorbidity is an important topic which has been inadequately addressed to date.

**Objective:**

We examined the extent of financial distress among persons with multimorbidity, using data from the 2017 Behavioral Risk Factor Surveillance System (BRFSS).

**Design:**

Cross-sectional, population-based study.

**Participants:**

Adults ages ≥ 18 years with multimorbidity.

**Main measures:**

Low income and selected social determinants of health that are indicators of financial distress.

**Key results:**

Multimorbidity was more common among those with a household income of less than $15,000 per year (P < 0.001) and among those who were 65 years of age or older (P < 0.001). There was an approximately linear increase in the percentage of individuals who had a household income of less than $15,000 or $25,000 per year with increasing number of morbidities. About one-quarter of individuals who had five or more morbidities had a household income of less than $15,000 per year as compared with 4.49% of individuals with no morbidities (P < 0.001). For all of the social determinants of health examined (Couldn’t pay bills, didn’t have money for food, didn’t have money for balanced meals, didn’t have enough money to make ends meet, and felt this kind of stress), there was an approximately linear increase in the percentage of individuals with an indicator of financial distress with increasing number of morbidities. Further research is needed examining the prevalence and correlates of financial distress in this population as well effective strategies for ameliorating its impact on the health and wellbeing of these persons.

## 1 Introduction

Over the past two decades, a sizeable literature has documented the problem of financial distress (financial hardship due to the high cost of health care) among persons with cancer and offered potential solutions (Zafar, 2016, Coughlin and Dean, 2019, Coughlin et al., 2020, Sadigh et al., 2019, Yezefski et al., 2018, Kircher et al., 2019, Lentz et al., 2018). More recently, authors have considered financial distress among persons with cardiovascular disease (CVD) (Kuehn, 2019, Khera et al., 2020, Khera et al., 2018, Valero-Elizondo et al., 2019). The high costs of care for chronic diseases such as cancer and CVD may lead to financial stress and have negative effects on the well-being of patients and their families (Desai and Gyawali, 2020, Carpenter et al., 2015 Jul 1, Banthin et al., 2008 Jan, Assari et al., 2019 Jan). Financial distress can contribute to worry, anxiety, and depression (Khera et al., 2020). Persons with CVD who are affected by financial distress are less likely to adhere to their medications (Khera et al., 2020). Among persons with cancer, financial distress has been shown to be associated with decreased treatment adherence, poorer quality of life, and worse survival (Shankaran et al., 2018). Persons with cancer may struggle to pay out-of-pocket expenses due to the high expenses incurred, the medical debt, or loss of work (Dean et al., 2018, Pisu et al., 2010). Women, younger persons, racial and ethnic minorities, low-income persons, and those without health insurance have an increased risk of financial distress (Carrera et al., 2018, Jagsi et al., 2014, Wheeler et al., 2018).

People with multimorbidity have higher health care costs which can create a financial burden (Larkin et al., 2021). A systematic review of multimorbidity cost-of-illness studies found that multimorbidity was always associated with higher out-of-pocket costs compared with non-multimorbidity (Wang et al., 2018). In addition, multimorbidity has been found to be associated with 5 to 10 times higher out-of-pocket costs for medications than no chronic conditions (Sum et al., 2018). Multimorbidity has also been associated with medicine nonadherence (Laba et al., 2020).

According to the World Health Organization, multimorbidity is the coexistence of two or more chronic conditions, where each must be a non-communicable disease, a mental health disorder, or an infectious disease of long duration (World Health Organization, 2016). The concept of multimorbidity is distinct from comorbidity because there is no primary condition or index condition from the perspective of the patient and the healthcare provider (Making more of multimorbidity, 2018, Tugwell and Knottnerus, 2019). Chronic conditions commonly included in studies of multimorbidity include CVD, stroke, hypertension, diabetes, chronic obstructive pulmonary disease (emphysema, chronic bronchitis), asthma, chronic kidney disease, arthritis, cancer, musculoskeletal disorders (e.g., arthritis, fractures), obesity, and chronic neurological and psychiatric conditions such as cognitive impairment and depression (King et al., 2018).

We examined the extent of financial distress among persons with multimorbidity, using data from the 2017 Behavioral Risk Factor Surveillance System (BRFSS). Of particular interest were associations between multimorbidity and low income, and with selected social determinants of health that are indicators of financial distress.

## 2 Methods

BRFSS is a cross-sectional telephone-based survey of U.S. residents 18 years of age and older which collects information about health-related risk factors, chronic health conditions, and use of preventive services (CDC Behavioral Risk Factor Surveillance Survey, 0000). Social determinants of health (World Health Organization, 0000) data were available for twelve states (Florida, Georgia, Iowa, Massachusetts, Minnesota, Mississippi, New Hampshire, Pennsylvania, Utah, West Virginia, Wisconsin, and Wyoming). The mean and median combined (landline and cell phone) response rates for this group of states were 47.23 and 46.25 percent respectively. To assess the relationship between financial distress and multi-morbidity, we first examined the distribution of respondents' household income categories as reported in the BRFSS (< $15,000, $15,000 to $24,999, $25,000 to $34,999, $35,000 to $ 49,000, and >= $50,000) by number of morbidities (0, 1, 2, 3, 4, and 5 + ). We further assessed the relationship with respect to selected social determinants of health. Respondents were asked, “During the last 12 months, was there a time when you were not able to pay your mortgage, rent, or utility bills?” (no, yes). They were also asked whether the following statements were often true, sometimes true, or never true: “The food that I bought just didn’t last, and I didn’t have money to get more.” And “I couldn’t afford to eat balanced meals.” Respondents were also asked, “In general, how do your finances usually work out at the end of the month? Do you find that you usually [don’t] have enough money to make ends meet? (no, yes). In addition, they were asked, “Stress means a situation in which a person feels tense, restless, nervous, or anxious, or is unable to sleep at night because his/her mind is troubled all the time. Within the last 30 days, how often have you felt this kind of stress?” (None of the time, A little/some of the time, All/most of the time).

There is currently no universally accepted definition of multimorbidiy. Based upon our literature review (Making more of multimorbidity, 2018, Tugwell and Knottnerus, 2019, King et al., 2018), we defined multimorbidity as two or more of the following chronic conditions: heart attack (myocardial infarction); angina or coronary heart disease; stroke; cancer (other than skin cancer); chronic obstructive pulmonary disease, emphysema or chronic bronchitis; arthritis, gout, lupus, or fibromyalgia; depressive disorder; kidney disease; and diabetes.

All analyses were conducted with Stata version 14.0 and used BRFSS sampling weights to adjust for the complex sampling design. The listwise deletion approach was applied to handle missing data. We first examined the prevalence of multimorbidity by demographic and socioeconomic factors. We then examined the frequency of individuals by number of morbidities and selected social determinants of health that are indicators of financial distress. We estimated the share of respondents unable to pay bills, didn't have money for food, balanced meals, and making ends meet, and felt associated stress; and performed two-sample t-tests on the equality of means to compare respondents with multi-morbidity with those of respondents with one or zero morbidity.

## 3 Results

Around one in every five individuals in our sample (N = 103,186) had more than one morbidity. Arthritis (74.44%, N = 20717) was the most common morbidity condition among individuals with multiple morbidities, followed by depressive disorders (49.58%, N = 12923), diabetes (34.62%, N = 9768), cancer (other than skin cancer) (24.43%, N = 7104). Among persons<65 years of age, multimorbidity was more common among women than men (DF = 68092, t-statistic = 10.16, P < 0.001) (Table1). Multimorbidity was more common among those with a household income of less than $15,000 per year (DF = 85226, t-statistic = 17.19, P < 0.001) and among those who were 65 years of age or older (DF = 102745, t-statistic = 44.85, P < 0.001) (Table 1).

**Table 1:** Prevalence of multimorbidity by demographic and socioeconomic factors.

	All	18–49	50–64	65+
	(%)			
**Sex**				
Male	18.56	6.44	24.64	42.82
	[10949]	[1351]	[3579]	[6019]
Female	23.19	10.20	30.80	41.92
	[16462]	[2447]	[5385]	[8630]
**Race**				
White	23.18	9.34	27.95	42.15
	[22832]	[2885]	[7197]	[12750]
Black	18.06	7.09	29.91	44.78
	[1920]	[2 7 1]	[7 8 6]	[8 6 3]
Hispanic	13.79	6.00	21.73	44.30
	[9 9 5]	[3 0 8]	[3 7 7]	[3 1 0]
Other	16.46	7.31	31.50	38.94
	[1673]	[3 3 4]	[6 0 6]	[7 3 3]
**Education**				
No education	20.00	3.48	13.89	76.38
	[46]	[5]	[12]	[29]
Elementary	33.66	10.34	44.56	53.30
	[8 3 1]	[91]	[2 6 8]	[4 7 2]
Some high school	29.58	12.78	42.16	49.86
	[1968]	[3 1 7]	[7 3 7]	[9 1 4]
High school graduate	23.05	8.83	30.74	42.94
	[8738]	[1133]	[2909]	[4696]
Some college	21.34	9.63	28.49	44.05
	[8402]	[1349]	[2817]	[4236]
College graduate	13.94	4.86	17.51	34.16
	[7345]	[8 9 0]	[2190]	[4265]
**Income**				
< $15,000	38.96	19.98	57.06	57.42
	[3830]	[6 8 7]	[1653]	[1490]
$15,000 to $24,999	27.86	13.04	42.22	45.08
	[5379]	[7 9 8]	[1743]	[2838]
$25,000 to $34,999	25.93	9.68	32.75	47.13
	[2927]	[3 5 3]	[8 1 8]	[1756]
$35,000 to $49,999	19.75	7.42	25.11	41.18
	[3268]	[4 0 5]	[9 4 1]	[1922]
≥ $50,000	13.71	5.15	18.01	35.27
	[7266]	[1067]	[2542]	[3657]
**All**	20.94	8.30	27.82	42.32
	[27420]	[3798]	[8966]	[14656]
*Two morbidities*	11.74	5.79	14.92	21.86
	[15092]	[2582]	[4826]	[7684]
*Three morbidities*	5.49	1.68	7.56	11.94
	[7166]	[8 1 5]	[2416]	[3935]
*Four morbidities*	2.24	0.56	3.12	5.11
	[3072]	[2 4 9]	[1004]	[1819]
*Five plus morbidities*	1.48	0.28	2.21	3.41
	[2090]	[1 5 2]	[7 2 0]	[1218]
Sample Size	103,186	37,871	30,691	34,624

Note: Estimates are obtained using complex survey weights of the 2017 BRFSS for the following twelve states – FL, GA, IA, MA, MN, MS, NH, PA, UT, WV, WI, and WY, for which social determinants of health information were available. Number of observations are in square brackets. Multimorbidity refers to having two or more of the following chronic conditions - heart attack/myocardial infarction; angina or coronary heart disease; stroke; cancer (other than skin cancer); COPD, emphysema or chronic bronchitis; arthritis, gout, lupus, or fibromyalgia; depressive disorder; kidney disease; and diabetes.

Table 2 shows the distribution of individuals by number of morbidities (Zafar, 2016, Coughlin and Dean, 2019, Coughlin et al., 2020, Sadigh et al., 2019) by income and selected social determinants of health. There was an approximately linear increase in the percentage of individuals who had a household income of less than $15,000 per year with increasing number of morbidities (Table 2). About one-quarter of individuals who had five or more morbidities also had a household income of less than $15,000 per year as compared with 4.49% of individuals with no morbidities (DF = 102745, t-statistic = 10.71, P < 0.001). A similarly approximately linear increase with increasing number of morbidities was seen for individuals who had a household income of $15,000 to $24,999 per year (Table 2). For all of the social determinants of health examined (Couldn’t pay bills, didn’t have money for food, didn’t have money for balanced meals, didn’t have enough money to make ends meet, and felt this kind of stress), there was an approximately linear increase in the percentage of individuals with an indicator of financial distress with increasing number of morbidities (Table 2). For example, almost one-fifth of individuals who couldn’t pay their mortgage, rent, or utility bills in the last 12 months had five or more morbidities as compared with 4.89% of individuals with no morbidities (DF = 87808, t-statistic = 8.70, P < 0.001).

**Table 2:** Frequency of individuals by number of morbidities and selected social determinants of health.

	Number of Morbidities					
	0	1	2	3	4	5+
	N (%)					
**Income**						
*(Sample size = 103186)*						
< $15,000	2103	1973	1583	1125	589	533
	(4.49)	(6.83)	(10.49)	(15.7)	(19.17)	(25.5)
$15,000 to $24,999	4975	3895	2626	1516	738	499
	(10.61)	(13.49)	(17.4)	(21.16)	(24.02)	(23.88)
$25,000 to $34,999	3659	2709	1575	801	333	218
	(7.8)	(9.38)	(10.44)	(11.18)	(10.84)	(10.43)
$35,000 to $49,999	5397	3638	1905	855	313	195
	(11.51)	(12.6)	(12.62)	(11.93)	(10.19)	(9.33)
≥ $50,000	22,911	11,737	4819	1619	545	283
	(48.87)	(40.64)	(31.93)	(22.59)	(17.74)	(13.54)
**Couldn't pay bills**						
*(Sample size = 88249)*						
No	36,811	23,059	11,845	5391	2232	1445
	(94.69)	(91.8)	(89.03)	(84.54)	(82.24)	(77.73)
Yes	1900	1959	1410	957	472	401
	(4.89)	(7.8)	(10.6)	(15.01)	(17.39)	(21.57)
**Didn't have money for food**						
*(Sample size = 88016)*						
Never	35,022	21,888	11,018	4801	1933	1193
	(90.38)	(87.35)	(83.02)	(75.43)	(71.3)	(64.21)
Sometimes	2763	2257	1496	989	481	387
	(7.13)	(9.01)	(11.27)	(15.54)	(17.74)	(20.83)
Often	689	748	656	512	268	265
	(1.78)	(2.98)	(4.94)	(8.04)	(9.89)	(14.26)
**Didn't have money for balanced meals**						
*(Sample size = 87973)*						
Never	34,359	21,383	10,785	4688	1854	1116
	(88.71)	(85.37)	(81.3)	(73.68)	(68.44)	(60.13)
Sometimes	3068	2404	1578	969	495	385
	(7.92)	(9.6)	(11.9)	(15.23)	(18.27)	(20.74)
Often	1045	1092	798	644	332	342
	(2.7)	(4.36)	(6.02)	(10.12)	(12.26)	(18.43)
**Didn't have enough money to make ends meet**						
*(Sample size = 87892)*						
No	36,069	22,599	11,408	5067	2055	1335
	(93.23)	(90.3)	(86.06)	(79.69)	(75.89)	(71.93)
Yes	1624	1783	1468	1099	576	478
	(4.2)	(7.12)	(11.07)	(17.29)	(21.27)	(25.75)
**Felt this kind of stress**						
*(Sample size = 87737)*						
None of the time	19,081	10,847	5211	2200	849	445
	(49.43)	(43.4)	(39.36)	(34.67)	(31.4)	(Sum et al., 2018)
A little/ some of the time	17,166	11,092	5748	2697	1104	751
	(44.47)	(44.38)	(43.41)	(42.51)	(40.83)	(40.51)
All/ most of the time	2131	2911	2186	1394	714	642
	(5.52)	(11.65)	(16.51)	(21.97)	(26.41)	(34.63)

Note: Estimates are obtained using data from the 2017 BRFSS for the following twelve states – FL, GA, IA, MA, MN, MS, NH, PA, UT, WV, WI, and WY, for which social determinants of health information were available. The following chronic conditions are considered - heart attack/myocardial infarction; angina or coronary heart disease; stroke; cancer (other than skin cancer); COPD, emphysema or chronic bronchitis; arthritis, gout, lupus, or fibromyalgia; depressive disorder; kidney disease; and diabetes. Column totals (in parenthesis) for certain determinants may not add up to 100 due to missing values.

## 4 Discussion

The results of this study indicate that the number of individuals with an annual income of less than $25,000 increases with the number of morbidities, and that a sizeable percentage of individuals with multimorbidity have difficulty paying bills or having enough food to eat.

There are several reasons why discussions of financial distress among persons with individual diseases (e.g., cancer or prior myocardial infarction) should be extended to encompass the care of persons with multimorbidity. First, low-income persons have a greater risk of multimorbidity (Pathirana and Jackson, 2018). Additionally, low socioeconomic status is associated with the early onset of multimorbidity (Mazza and Mitchell, 2017). Residents of socioeconomically deprived areas develop multimorbidity 10 to 15 years earlier than those from more affluent areas (Mazza and Mitchell, 2017). Second, multimorbidity is costly both to healthcare systems and to individual patients (Making more of multimorbidity, 2018). Persons with multimorbidity are frequently prescribed multiple medications and are disproportionately burdened financially due to their complex healthcare needs (King et al., 2018). Third, although the prevalence of multimorbidity increases with age and multimorbidity is associated with geriatric syndromes such as frailty (Forman et al., 2018, Chi, 2017), there are more people living with multimorbidity among those younger than 65 years as they represent a larger segment of the population (Forman et al., 2018).

To assist patients with multimorbidity deal with financial distress, financial distress screening can be incorporated into clinic workflow and financial counselors can be employed. Implementing solutions at different levels of the healthcare system is essential (Desai and Gyawali, 2020). At the hospital level, measures that hospitals can take to address financial distress include cost transparency and provision of financial counselors (Desai and Gyawali, 2020). An additional benefit of on-site patient financial counselors would be in supporting physicians and advanced practice providers in ensuring that individuals are capable of obtaining medications that have been prescribed for them. A multidisciplinary approach involving nurses and social workers may provide additional expertise in mitigating financial distress as experienced by patients. Social workers may help their patients locate grants from national or local community programs (Chi, 2017). Persons with multimorbidity may not be knowledgeable about insurance concepts such as deductibles and coinsurance (Zafar, 2016). Referrals to financial assistance programs and community resources can be useful. Educational interventions should focus on educating patients on the basics of health insurance and costs they may face during treatment, as well as patient assistance programs (Zafar, 2016). Continued efforts aimed at reforming health care in the United States are essential to mitigate the problem of financial distress among persons with multimorbidity.

With respect to limitations, the study was cross-sectional in nature. The comparisons across groups were subject to limitations of the *t*-test. We lacked information about how long participants had been low-income. In addition, no information was available about whether job loss impacted financial stress.

Further research is needed investigating the prevalence and correlates of financial distress in this population. Greater efforts should also be directed to creating and implementing effective strategies aimed at ameliorating its adverse impacts on the health and wellbeing of these persons.

## Declaration of Competing Interest

The authors declare that they have no known competing financial interests or personal relationships that could have appeared to influence the work reported in this paper.
